# A Novel Magnetoelastic Immunosensor for Ultrasensitively Detecting Carcinoembryonic Antigen

**DOI:** 10.1186/s11671-018-2632-0

**Published:** 2018-08-29

**Authors:** Yixia Zhang, Xing Guo, Lu Fan, Qiang Zhang, Shengbo Sang

**Affiliations:** 1 Institute of Applied Mechanics and Biomedical Engineering & National Demonstration Center for Experimental Mechanics Education, College of Mechanics, Taiyuan University of Technology, Taiyuan, 030024 China; 2 Micro-Nano System Research Center & Key Lab of Advanced Transducers and Intelligent Control System of the Ministry of Education, College of Information and Computer, Taiyuan University of Technology, Taiyuan, 030024 China

**Keywords:** Magnetoelastic immunosensor, Gold nanoparticles, Resonant frequency, Antibody, CEA

## Abstract

A novel wireless immunosensor is developed for the ultra-sensitive detection of carcinoembryonic antigen. The optimum dimension of the microchips, as magnetoelastic sensitive units, was evaluated by simulation and experiments. The unique effects signal amplification and biocompatibility of gold particles contribute to the stability and sensitivity of the sensor. Furthermore, to enhance sensitivity, the working concentrations of antibody and BSA are selected to be 50 mg/mL and 0.1%, respectively. Atom force microscope imaging sheds light on the biological analysis. The Nano-magnetoelastic immunosensor exhibits a linear response to the logarithm of carcinoembryonic antigen (CEA) concentrations ranging from 0.1 to 100 ng/mL, with a detection limit of 2.5 pg/mL. The designed biosensor has merits of excellent stability and sensitivity towards CEA.

## Background

Cancer is one of the fatal diseases in the world [1]. The cancer in patients can be clinically detected when the concentration of tumor biomarkers reaches up to a certain amount in serum [2]. Therefore, it is quite necessary to achieve sensitive, fast, and accurate assays for tumor markers, which provide an effective strategy for diagnosis of cancer [3]. Carcinoembryonic antigen (CEA) is of a family of cell surface glycoproteins with a molecular weight of 180∼200 kDa. It was firstly discovered in human colon cancer tissue in 1965 [4, 5]. CEA usually presents at very low levels (0~5 ng/mL) in the blood of healthy adults [6]. Generally, an abnormal level of CEA may be regarded as a sign of cancer, such as gastric carcinoma [7], pancreatic carcinoma [8], colorectal carcinoma [9], lung carcinoma [10], and breast carcinoma [11]. It means that CEA could be used as a tumor biomarker. Monitoring the CEA level in blood could be utilized to pre-warn, screen, and diagnose cancers. Meanwhile, the CEA can also be used for follow-up research of those who have been treated clinically. The sensitivity of CEA to tumor recurrence is over 80%, which is earlier than clinical and pathological examination. So, the continuous observation of the CEA provides an important basis for diagnosis and prognosis of the curative effects [12].

Biosensors respond to specific recognitions of biological molecular output measurable signals by some discipline, allowing quick responses, high sensitivity, and low cost. Recently, immunological biosensors have been intensively studied, such as enzymetic immunoassay [13], fluoro-immunoassay [14], and electrochemical immunoassay [15–17]. Due to its excellent specificity and sensitivity, immunosensors provided promising means for the analysis of tumor biomarkers, even when the target compounds are in very low concentrations [18–21].

The nanotechnology is providing novel methods for the application of nanoparticles (NPs) in biosensing technology. Metal NPs exhibit many special characteristics, which provide remarkable platforms for interfacing bio-recognition elements [22, 23]. Immunoassays based on NPs have attracted great attention for the researchers [24–26]. The magnetoelastic biosensors are not affected by ambient temperature and pH with high response sensitivity. Therefore, in this study, we proposed a magnetoelastic immunoassay method based on gold nanoparticles (AuNPs) and magnetoelastic microchips. An immunosensor was successfully developed for detecting CEA biomarkers.

## Results and Discussion

**Fig. 1:**
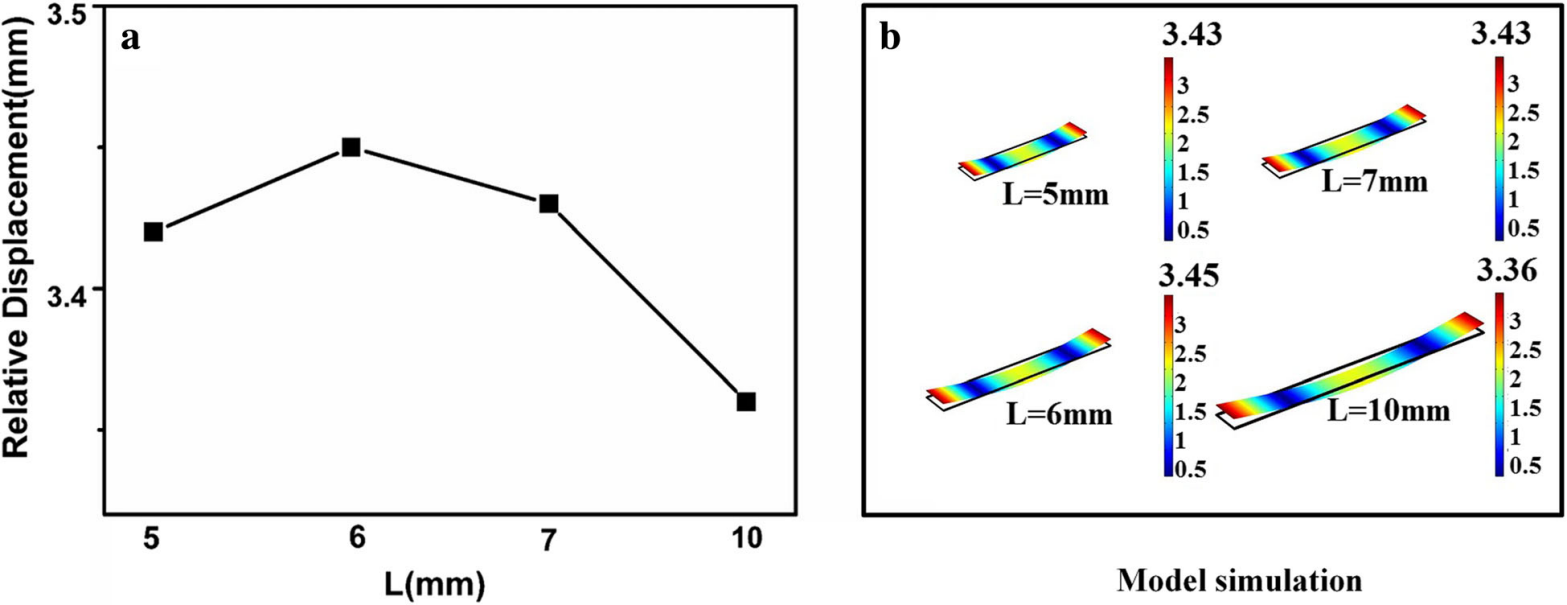
Optimum length of the ME chip. **a** The relative displacement is different with the variation of length. **b** Simulation was used to optimize the length of the chip

In view of the ribbon-like shape of the magnetoelastic (ME) microchip, the magnetic permeability is greatest along its length [27]. The preliminary results have shown that the optimum width and thickness of the ME chip were 1 mm and 28 μm, respectively [28]. Simulation was used to optimize the length of the chip, as demonstrated in Fig. 1b.

The relative displacement is different with the variation of length in Fig. 1a. The maximum relative displacement is obtained when the length is 6 mm under the first-order modal analysis. It means the theoretically highest sensitivity. Therefore, the optimum dimensions of the chip were designed as 6 mm × 1 mm × 28 μm in this paper.

**Fig. 2:**
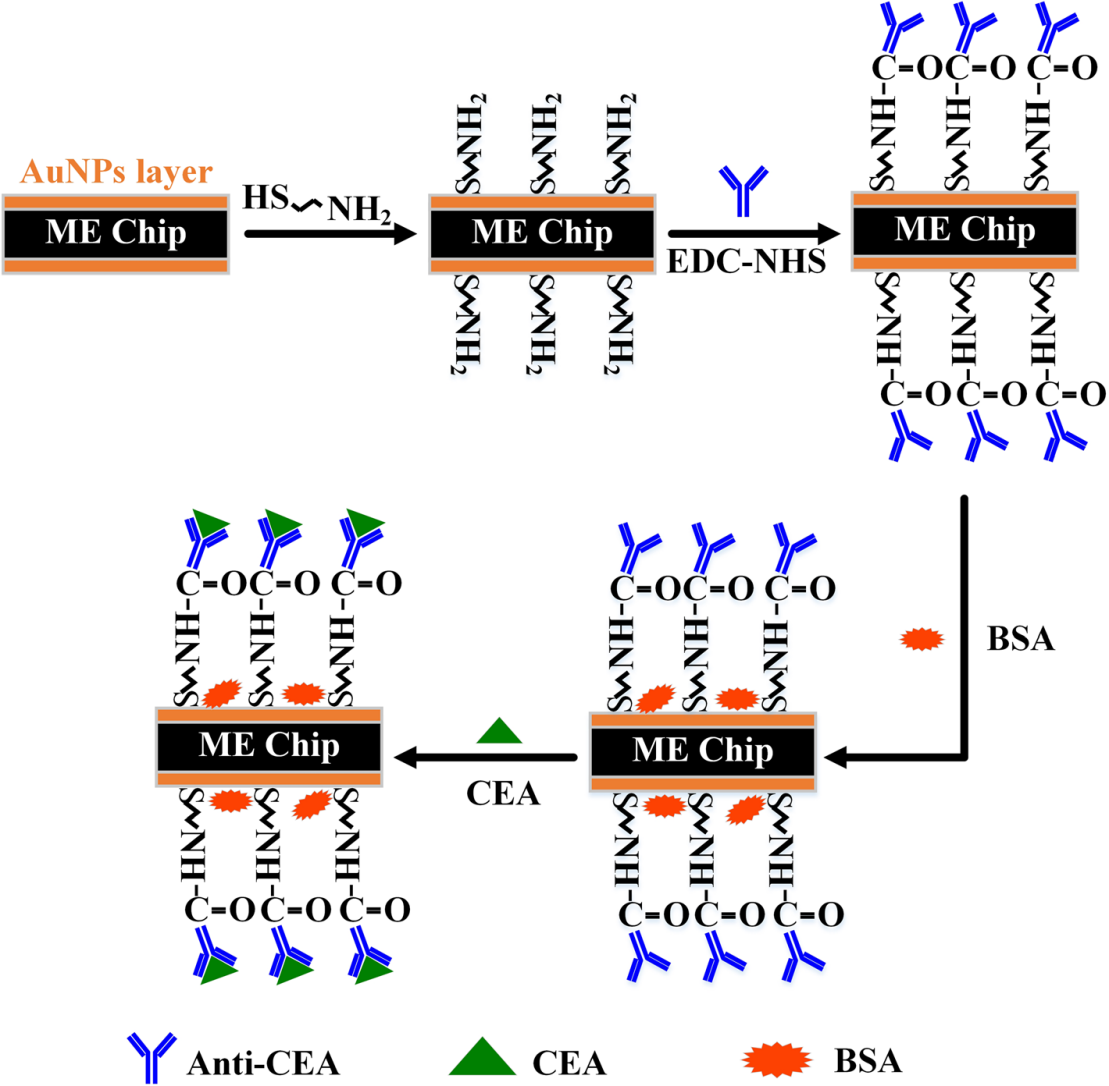
Scheme of the constructed Nano-ME biosensor

**Fig. 3:**
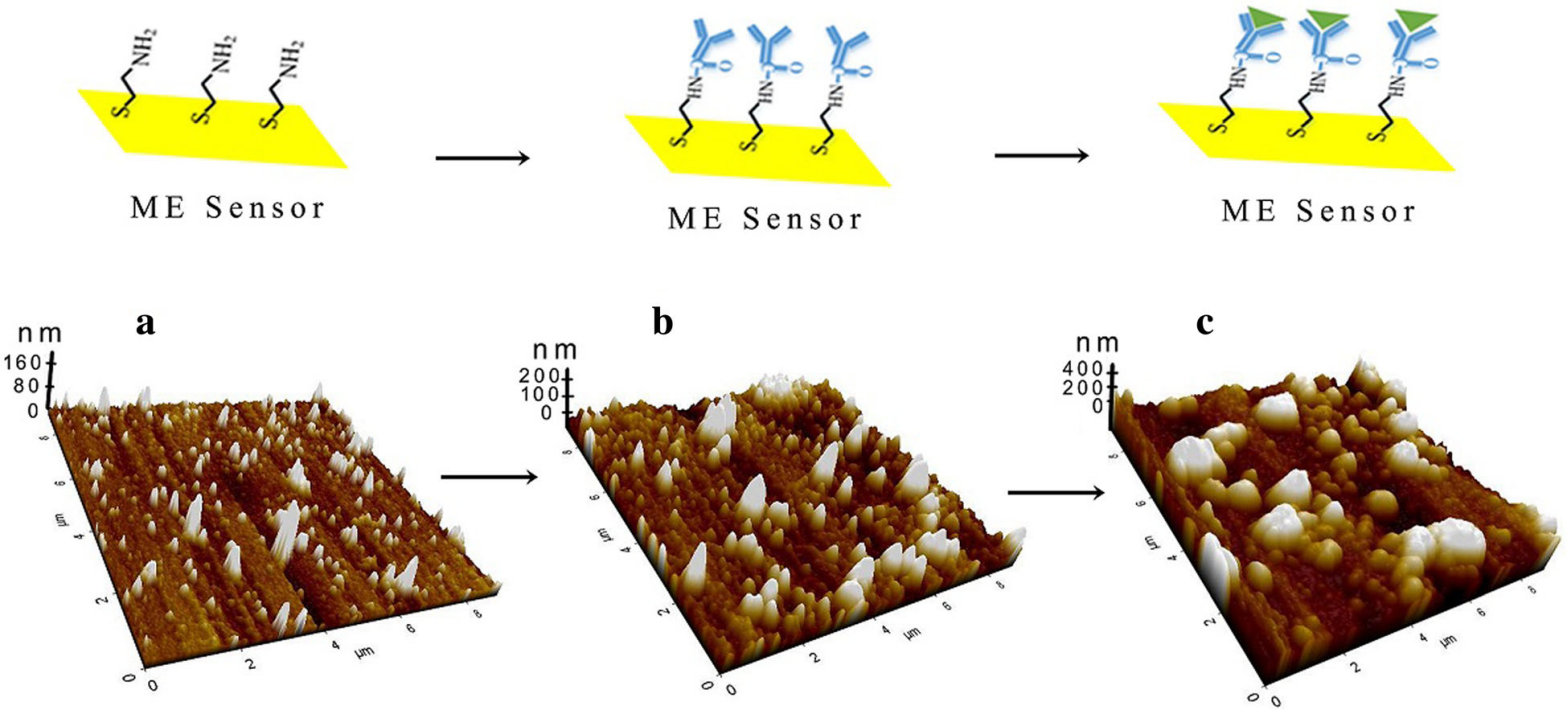
AFM images of SAM layer (**a**). CEAAb-SAM layer (**b**). Complex of CEA-CEAAb(**c**)

A schematic diagram of the Nano-ME biosensor is illustrated in Fig. 2. Firstly, the Nano-ME chip was chemically treated by cysteine to fabricate the self-assembling molecular (SAM) films on the surface, as a functional layer for immobilization of CEAAb. Then, bovine serum albumin (BSA) promotes the performances of CEAAb by reducing non-specific binding and steric hindrance. Atom force microscope (AFM) images were carried out for observing the surface morphology of the chip. As indicated in Fig. 3a, the thickness of SAM layer was 120 nm. The imaging in Fig. 3b reveals that the CEAAb was covalently attached to SAM layer with increasing roughness. It was clearly displayed in Fig. 3c that the CEA was specifically recognized and effectively combined, with an approximate height of 200 nm and larger size.

**Fig. 4:**
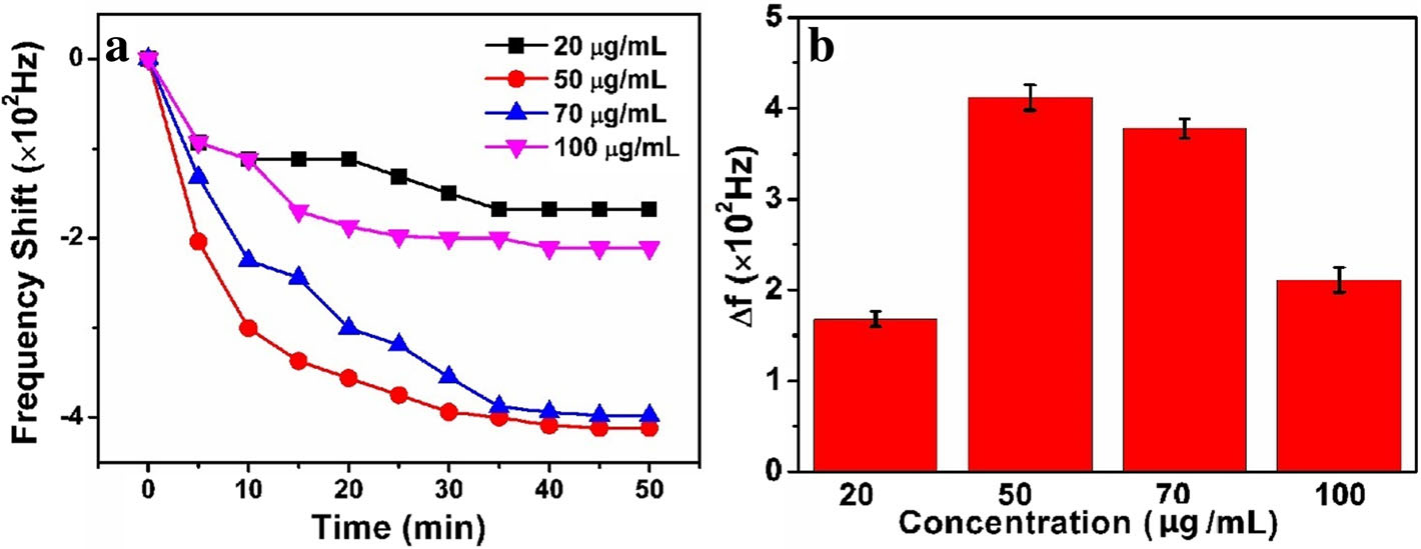
**a** The curve of frequency response versus CEAAb. **b** Frequency histogram

In a certain dimension of the chip, the concentration of antibody is an important factor related to the sensitivity of the immunosensor. Therefore, it was necessary for evaluating the response signals of different concentrations of CEAAb (20, 50, 70, and 100 μg/mL, as shown in Fig. 4a). The results show that the optimum response was obtained at approximately 448 Hz (Fig. 4b), when the concentration of CEAAb is 50 μg/mL. If the concentration of CEAAb increased to 70 μg/mL, the response began to decline due to the steric hindrance and the electrostatic repulsion [29].

**Fig. 5:**
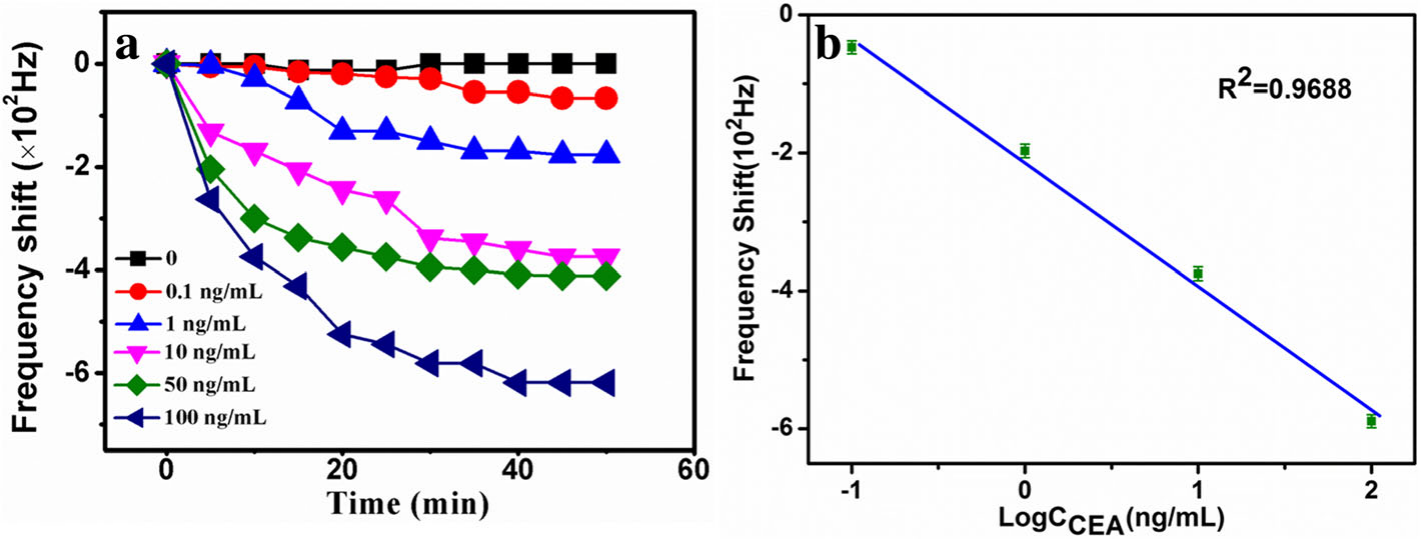
Real-time response (**a**) and fitting curves (**b**) of the biosensor versus CEA

In principle, the CEA is specifically recognized with antibody, which leads to the decrease of the response frequency. Figure 5a shows the real-time response curve of the immunosensor towards CEA. Meanwhile, we acquire a linear fitting curve in Fig. 5b.

Generally, the stable response of the sensor was achieved at 40 min (Fig. 5a). The change of the resonance frequency was recorded with corresponding concentrations of CEA. The change of Hz is linearly dependent on the logarithm of CEA concentrations ranging from 0.1 to 100 ng/mL (*R*^2^ = 0.9688), with the detection limit of 2.5 pg/mL (Fig. 5b). To our knowledge, the linear range and the detection limit are obviously lower than those of the previous methods [28]. The results demonstrated that a wireless and highly sensitive method towards CEA was successfully established.

## Conclusions

In this contribution, a Nano-ME immunosensor for highly sensitive detection of CEA was successfully developed based on ME chip. AuNPs and BSA effectively improved the sensitivity and stability. The proposed Nano-ME immunosensor exhibits wide determination ranges of CEA from 0.1 to 100 ng/mL with a low detection limit of 2.5 pg/mL. Therefore, the accurate determination of CEA by the as-prepared immunosensor was achieved with satisfactory results. Benefiting from its specificity, simplicity, and reproducibility, the proposed platform shows a promising application in the development of non-invasive cancer detection.

## Methods

Under the time-varying magnetic field, the ME microchip vibrates along the length. In the modulated magnetic field to make the ME microchip vibrate, the energy of the magnetic field is converted into elastic potential energy to reach the maximum value. Due to the shape of the ribbon-like sensor chip, the magnetic permeability is greatest along its length; hence, an incident magnetic field generates longitudinal vibrations in the sensor from almost any orientation except normal to the basal plane of the sensor. Given by Eq. (1):

1 $$ {f}_0=\frac{1}{2L}\sqrt{\frac{E}{\rho \left(1-{\nu}^2\right)}} $$

where *E* denotes modulus of elasticity, *v* is Poisson’s ratio, *ρ* is the density of the sensor material, and *L* is the longitudinal dimension of the chip. When the test temperature, humidity, and other environmental parameters are constant, the resonance frequency change of the magnetoelastic sensor sensitively depends only on the mass change (△*m*) on its surface, as given by Eq. (2)

2 $$ \frac{\triangle f}{\triangle m}=-\frac{f_0}{2M} $$

Based on Eq. (2), the change of resonance frequency is proportional to the amount of CEA. Therefore, the CEA concentrations can be achieved by the change of frequency, where *f*_0_ is the initial resonance frequency, *M* is the initial mass, △*m* is the mass change, and △*f* is the shift in the resonance frequency of the sensor. Equation 2 shows that sensor sensitivity (△*f*/△*m*) is inversely proportional to initial magnetoelastic mass (M) of the sensor. Sensors with smaller physical dimensions have a lower initial mass resulting in higher sensitivity. The negative sign in the equation represents a decrease in frequency (△*f*) to an addition of non-magnetoelastic mass (△*m*) on the sensor. Hence, binding of the target organisms onto the biosensor surface causes a mass increase with a corresponding decrease in fundamental resonance frequency.

Magnetoelastic bases of Metglas alloy 2826MB (Fe40Ni38Mo4B18) were processed by Honey well Corporation (Morristown, NJ, USA). CEA, CEA antibody, bovine serum albumin (BSA, 99%), and phosphate buffered saline (PBS, pH = 7.4) were purchased from Sangon (Shanghai, China). Acetone, isopropanol, ethanol, 1-ethyl-3-carbodiimide (EDC), and *N*-hydroxysulfosuccinimide (NHS) were purchased from Sigma-Aldrich Corporation (Saint Louis, MO, USA). All other reagents were of analytical grade. The ultrapure water was obtained from Mill-Q system (Milli-pore, USA). AFM Park System (ND-100, Korea), Plasma (P3C, Shanghai, China), Gauss ohmmeter (GM500), ZNB Vector Network Analyzer (R&S, Germany), Laser cutter (AV3620A, Qingdao, China), and HT20 gauss meter (Hengtong, Shanghai) were used.

The alloy ME base was laser-cut to 6 mm × 1 mm × 28 μm microchips, then ultrasonically cleaned with acetone, isopropanol, ethanol, and deionized water for 5 min and dried with nitrogen. The activation of the surface modification of the cleaned microchips is processed by a plasma method. Both sides of the microchip were sputtered with chromium layer (100 nm), followed by coating with AuNP layer (40 nm) to fabricate Nano-ME chips. The Nano-ME chip deals with plasma with high purity oxygen (0.9999) and then immersed into 40 mM cysteamine solution and kept for 12 h at room temperature. After that, the Nano-ME chips were biologically modified and incubated with different concentrations of CEAAb for 1 h at 37 °C in the presence of 1-ethyl-3-carbodiimide (EDC) and *N*-hydroxysulfosuccinimide (NHS). The CEAAb was firstly activated with 10 mg/mL EDC and 10 mg/mL NHS. Finally, the Nano-ME chip, modified by CEAAb, was further conducted with 0.1% BSA for 30 min.

The Nano-ME biosensor was constructed as follows: a glass tube was wrapped by the coil and connected to a vector network analyzer. Meanwhile, adding magnetic field provided alternating current to make the coil produce alternating magnetic field. The resonant frequency of the Nano-ME biosensor can be obtained by a vector network analyzer. Different concentrations of CEA (0–100 ng/mL) were added into the test tube, and the frequency shift was recorded every 5 min until 40 min. After that, the Nano-ME chip was rinsed with PBS for AFM characterization.

## Abbreviations

AFM: Atom force microscope
AuNPs: Gold nanoparticles
BSA: Bovine serum albumin
CEA: Carcinoembryonic antigen
CEAAb: CEA antibody
EDC: 1-Ethyl-3-carbodiimide
Hz: Frequency
ME: Magnetoelastic
NHS: *N*-Hydroxysulfosuccinimide
PBS: Phosphate buffered saline
SAM: Self-assembling molecular
